# Application of Deep Learning Architectures for Accurate and Rapid Detection of Internal Mechanical Damage of Blueberry Using Hyperspectral Transmittance Data

**DOI:** 10.3390/s18041126

**Published:** 2018-04-07

**Authors:** Zhaodi Wang, Menghan Hu, Guangtao Zhai

**Affiliations:** Institute of Image Communication and Information Processing, Shanghai Jiao Tong University, Shanghai 200240, China; wangzhaodi@sjtu.edu.cn

**Keywords:** convolutional neural networks, hyperspectral transmittance image, internal mechanical damage detection, fruit quality detection, machine learning

## Abstract

Deep learning has become a widely used powerful tool in many research fields, although not much so yet in agriculture technologies. In this work, two deep convolutional neural networks (CNN), viz. Residual Network (ResNet) and its improved version named ResNeXt, are used to detect internal mechanical damage of blueberries using hyperspectral transmittance data. The original structure and size of hypercubes are adapted for the deep CNN training. To ensure that the models are applicable to hypercube, we adjust the number of filters in the convolutional layers. Moreover, a total of 5 traditional machine learning algorithms, viz. Sequential Minimal Optimization (SMO), Linear Regression (LR), Random Forest (RF), Bagging and Multilayer Perceptron (MLP), are performed as the comparison experiments. In terms of model assessment, k-fold cross validation is used to indicate that the model performance does not vary with the different combination of dataset. In real-world application, selling damaged berries will lead to greater interest loss than discarding the sound ones. Thus, precision, recall, and F1-score are also used as the evaluation indicators alongside accuracy to quantify the false positive rate. The first three indicators are seldom used by investigators in the agricultural engineering domain. Furthermore, ROC curves and Precision-Recall curves are plotted to visualize the performance of classifiers. The fine-tuned ResNet/ResNeXt achieve average accuracy and F1-score of 0.8844/0.8784 and 0.8952/0.8905, respectively. Classifiers SMO/ LR/RF/Bagging/MLP obtain average accuracy and F1-score of 0.8082/0.7606/0.7314/0.7113/0.7827 and 0.8268/0.7796/0.7529/0.7339/0.7971, respectively. Two deep learning models achieve better classification performance than the traditional machine learning methods. Classification for each testing sample only takes 5.2 ms and 6.5 ms respectively for ResNet and ResNeXt, indicating that the deep learning framework has great potential for online fruit sorting. The results of this study demonstrate the potential of deep CNN application on analyzing the internal mechanical damage of fruit.

## 1. Introduction

Blueberry is a small soft fruit with high economic value because of its excellent flavor, abundant nutrients, and potential anticancer attribute [1]. Nonetheless, when blueberries undergo postharvest operations, they are extremely susceptible to mechanical damage because of their soft texture [2]. Once the berries are damaged, the softening procedure will be accelerated and the risk of pathogen infection is elevated [3]. Afterwards, this will reduce the income of the blueberry industry and bring the possible hazard to the consumer [4]. Therefore, it is essential to leverage some effective strategies to remove the damaged berries for transportation and marketing.

Since the blueberry skin is composed of deep dark pigments, the pulp and other tissues under the skin is non-visible to the naked eye [5]. Hence, it is a challenge to utilize the RGB (Red Green Blue) imaging technique and human eye detection method to accurately screen out berries with mechanical damage underneath the skin. Moreover, for manual inspection by human eye, the procedure is time-consuming and error-prone. Nondestructive measurement techniques such as near infrared spectrograph [6], hyperspectral imager [7] and thermal imager [8], which can examine the internal state of the testing object, have been regarded as the alternative solutions to traditional measurement and analysis techniques. Among these techniques, hyperspectral imaging technique combining the advantages of both imaging and spectroscopic techniques has emerged as a powerful tool for the quality evaluation of food and agricultural products [9]. For the hyperspectral imaging system, there exist four imaging modes viz. reflectance, transmittance, interactance and scattering [10,11]. In the case of detection of internal damage, the hyperspectral transmittance imaging system has been reported as the most effective architecture for inspecting the insects in soybean [12] and parasites in shell-free cooked clam [13]. Zhang et al., validated the feasibility of hyperspectral transmittance imaging mode for quantifying blueberry bruises [14]. Hu et al., compared the performances of hyperspectral reflectance, transmittance and interactance imaging modes for detection of sightless blueberry damage [15]. The results demonstrated that the hyperspectral transmittance imaging mode was identified to be more sensitive to sightless blueberry damage than reflectance and interactance modes. For this reason, only hyperspectral transmittance data of blueberry was collected for data analysis.

In terms of secondary measurement approach, we should always find the suitable machine learning algorithm to address the specific problem. Over recent decades, most investigators in agricultural engineering used conventional machine learning algorithms such as artificial neural network [16] and support vector machine [17]. As one of the machine learning methods, deep learning has been reported as the first of 10 breakthrough technologies by MIT technology review [18]. Deep learning has dramatically promoted the development of many research domains such as speech recognition and object detection [19]. There is no doubt that the use of deep learning is an irresistible general trend for the future of agriculture. Some investigators had attempted to apply deep learning in agricultural engineering. King reported that the integration of deep learning in sweet-pepper harvester software can give superior performance [20]. Liu et al. used convolutional neural network (CNN) for analyzing hyperspectral data, and their results indicated that the deep learning framework can give excellent performance for detection of defect regions on surface-defective cucumbers [21]. Jeon and Rhee also used CNN model to classify leaves, and the recognition rate was greater than 94% [22]. Similar CNN models were also used for tomato disease recognition [23] and melon lesion detection [24]. Sa et al. implemented Faster Region-based CNN (Faster R-CNN) model for fruit detection and achieved F1-score of 0.838, using two imagery modalities, i.e., color (RGB) and Near-Infrared (NIR) [25]. Bargoti et al. proposed a similar system to detect mangoes, almonds, and apples in orchards, resulting in F1-score more than 0.9 [26].

The contribution of this study is to introduce deep learning technique into the agricultural engineering practice of soft fruit damage detection based on hyperspectral transmittance image. Compared to traditional or manual methods, the implementation of deep learning will reduce time costs and improve detection accuracy.

In this work, we attempt to use two newly developed CNN architectures and hyperspectral transmittance data for the detection of blueberry damage underneath skin. The specific objectives of the current study are to: (1) acquire the hyperspectral transmittance data of damaged and sound blueberries using the in-house hyperspectral diffuse transmittance imaging system; (2) fine-tune two CNN architectures, Residual Network (ResNet) [27] and its improved version named ResNeXt [28], via the use of TensorFlow and adjust the data structure of blueberry dataset to meet the data requirements of CNN models; (3) validate the performances of these two proposed CNN architectures using k-fold validation; and (4) implement traditional machine learning models to complete the classification task and compare the results with that of CNN method.

## 2. Materials and Methods

### 2.1. Hyperspectral Transmittance Imaging System

**Figure 1:**
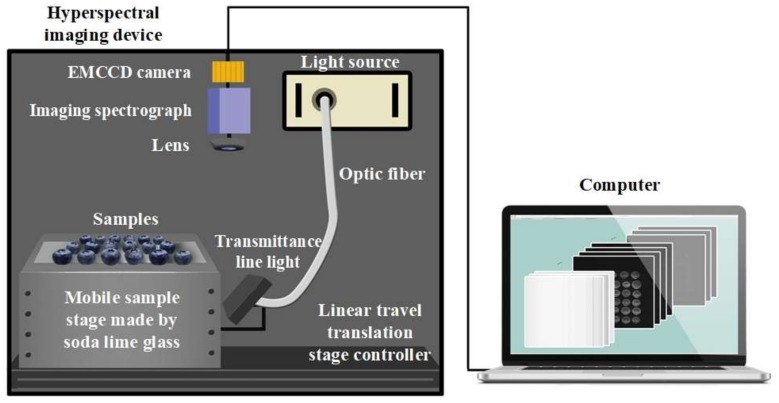
Schematic of hyperspectral transmittance imaging system.

Figure 1 shows the schematic of hyperspectral transmittance imaging system by Isuzu Optics Corp., Taiwan according to our design. This hyperspectral transmittance imaging system operates in a line-scanning mode, and consists of a spectrograph (Imspector V10E, Spectral Imaging Ltd., Oulu, Finland) combined with a 16-bit electron-magnifying charge-coupled detector (EMCCD) camera (Falcon EM285CL, Raptor Photonics Led., Northern Ireland, UK) and a C-mount lens (Xenoplan 1.4/17, Jos. Schneider Optische Werke GmbH, Bad Kreuznach, Germany), a line lights (9130-HT, Illumination Technologies, Inc., New York, NY, USA) assembling collector lenses (9560, Illumination Technologies, Inc., New York, NY, USA), and a mobile sample stage with a soda lime glass controlled by a linear travel translation stage controller (IRCP0076-1COMB, Isuzu Optics Corp., Taiwan). The spectral range of this hyperspectral imaging system is from 328.81 nm to 1113.54 nm with full width at half maximum of approximately 0.78 nm.

During hyperspectral cube acquisition, blueberry samples are placed on a mobile stage and move along the slide with the speed of 3 mm/s. To capture hypercube with high quality, the intensity of line light and exposure of EMCCD camera are set to 59 Klux and 30 ms, respectively.

### 2.2. Blueberry Database Description

A total of 737 blueberries were collected from Frutera San Fernando S.A., Chile from December 2014 to January 2015. The blueberries were stored at 4 °C and relatively humidity of about 85% after being transported to the lab in Shanghai, China. To guarantee the model robustness, only blueberries with little visible physical damage and sound surface were used for analysis.

**Figure 2:**
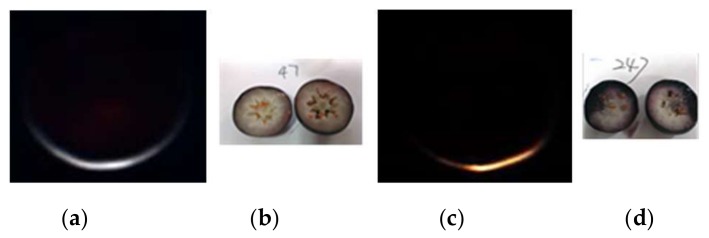
Hyperspectral transmittance images of the sound (**a**) and damaged (**c**) samples and their corresponding ground truth information ((**b**,**d**) for the sound and damaged samples, respectively). The presented hyperspectral transmittance images are generated by stacking sub-images at R, G and B wavelengths sequentially.

Since the internal mechanical damage of the blueberry was invisible, all berries were cut through the equator (Figure 2b,d) to ensure the ground truth information of the samples. When blueberries have not yet been cut, it is difficult to distinguish between the sound and the damaged with the naked eye and some traditional solutions. According to the damage degrees, the damaged areas consisting of more than 25% of the cut surface were classified as the damaged category. The database used in this study is composed of 557 blueberry samples, including 304 sound samples and 253 damaged samples.

**Figure 3:**
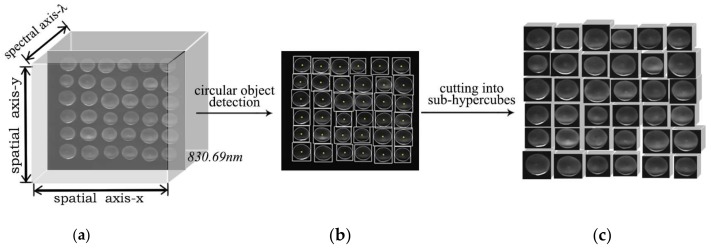
Data acquisition process. (**a**) Original hypercube; (**b**) Circular object detection based on morphological image processing; (**c**) The cut sub-hypercube.

Blueberry hyperspectral images were collected in bulk to imitate the batch data acquisition process in industrial production. Figure 3 shows the data acquisition process. In Figure 3a, the image at a typical wavelength (the transmittance value of the sample surface is relatively high, and here is 830.69 nm) is selected to locate the enclosing rectangle of each sample. After the position of each sample on the xOy plane is determined (Figure 3b), the original data is cut into sub-hypercubes for the following work.

**Figure 4:**
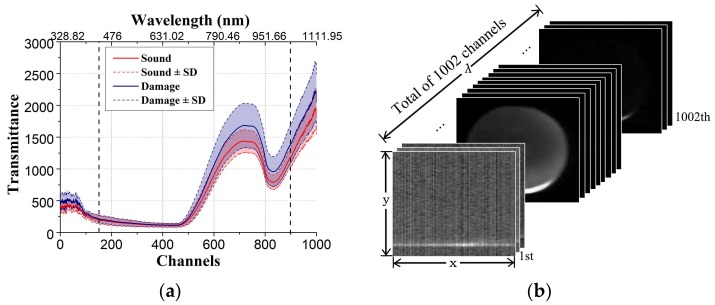
(**a**) Average transmittance extracted from each channel in hypercube; (**b**) Data structure of the original hyperspectral image cube. In (**b**), x, y and λ denote spatial x-axis, spatial y-axis, and spectral λ-axis, respectively.

Figure 4 shows the data structure of hyperspectral transmittance image cube. As shown in Figure 4a, it can be observed that there exists overlapped region in transmittance spectra of sound and damaged blueberries. Most traditional machine learning algorithms always apply the nonlinear models for identification of sound and damaged blueberries using spectral data. However, these spectral data cannot represent the structural alter caused by outside load force, and we need more representative image features. CNN extracts image features implicitly by convolutional layers, providing an excellent solution to obtain depth features from hypercube, allowing the establishment of a robust and reliable classifier. Figure 4b shows the data structure of hyperspectral transmittance image cube. The width and height of images vary from 100 to 130 pixels. Each image cube contains 1002 spectral channels, whose wavelengths vary from 328.82 nm to 1113.54 nm, with incensement of 0.72 nm to 0.81 nm.

### 2.3. CNN Models for Blueberry Damage Detection

#### 2.3.1. Data Preprocessing for CNN Models

A total of 1002 channels for the input of the convolution neural network is unreasonable, since excessive input data points will bring redundant parameters to be trained, which easily leads to overfitting. Therefore, the original data need to be subsampled. The unstable points in the original data are not conducive to model training. Besides, the data of the adjacent channels is similar and hence there exists redundancy (high linear relationship). According to Figure 4a, the average transmittance spectra locating on the first and last few channels fluctuate relatively large. Thus, these channels will not be included in the model training. Then, we choose the 150th channel to the 900th channel, and sub-sample with 5 spectral intervals. Finally, we obtain an image cube of 151 channels with a spectral range from 438.26 nm to 1032.08 nm. To reduce computation complexity, all the resulting images are further resized to the resolution of 32 × 32.

Although the size of our database is large enough for the traditional machine learning algorithms, it still cannot reach the requirement of deep learning. Thus, we conducted data augmentation. Each image is flipped vertically, flipped horizontally, rotated by 90°/180°/270°, and randomly cropped (resize the original image to the resolution of 128 × 128, then randomly take sub-images with the resolution of 100 × 100), respectively. The expanded sample size is seven times the sample size of the original training set. It should be noted that data augmentation is only implemented on the training set, not the testing set.

Then, we zero center every sample with specified mean and scale each sample by the specified standard deviation. The mean and standard deviation are evaluated per wavelength channel.

**Figure 5:**
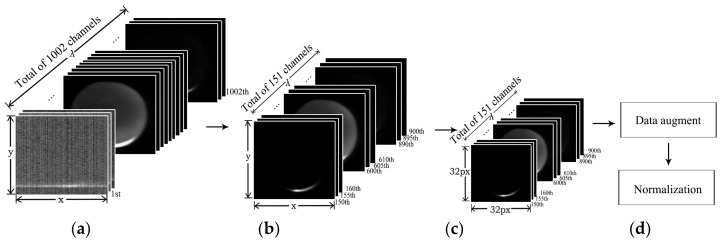
Data preprocessing for CNN models. (**a**) Original hypercube with 1002 channels of various spatial sizes; (**b**) Subsampled hypercube with 151 channels; (**c**) Resized to 32 px × 32 px to reduce computation cost; (**d**) Data augment and normalization.

Figure 5 presents the flow of hypercube preprocessing for CNN models. To sum up, the preprocessing procedure contains subsampling, image resizing, data augment and normalization.

In the aspect of model assessment, k-fold cross validation is used to demonstrate that the model performs well on different combinations of dataset. Blueberry samples are randomly grouped into 10 mutually exclusive subsets. Each time one of the subsets is chosen as the testing set, and the left (k − 1) subsets as training set. In this study, k is placed at 10. Thus, we obtain 10 groups of training/testing set, allowing 10 training processes.

#### 2.3.2. Model Fine-Tuning

In this section, we train the blueberry dataset with ResNet and ResNeXt, which are models proposed in recent years. These two deep frameworks were first developed for natural color image and had achieved great performance on the ImageNet dataset. Unlike the natural color images, hyperspectral images contain additional spectral dimension. Therefore, the number of filters in the convolutional layers are adjusted to make these two models appropriate for hyperspectral data structure. The parameters of the model used in this study are determined after repeated attempts to meet the requirements of high-dimensional data as input. The detailed descriptions about the model architectures are presented as follows.

**Figure 6:**
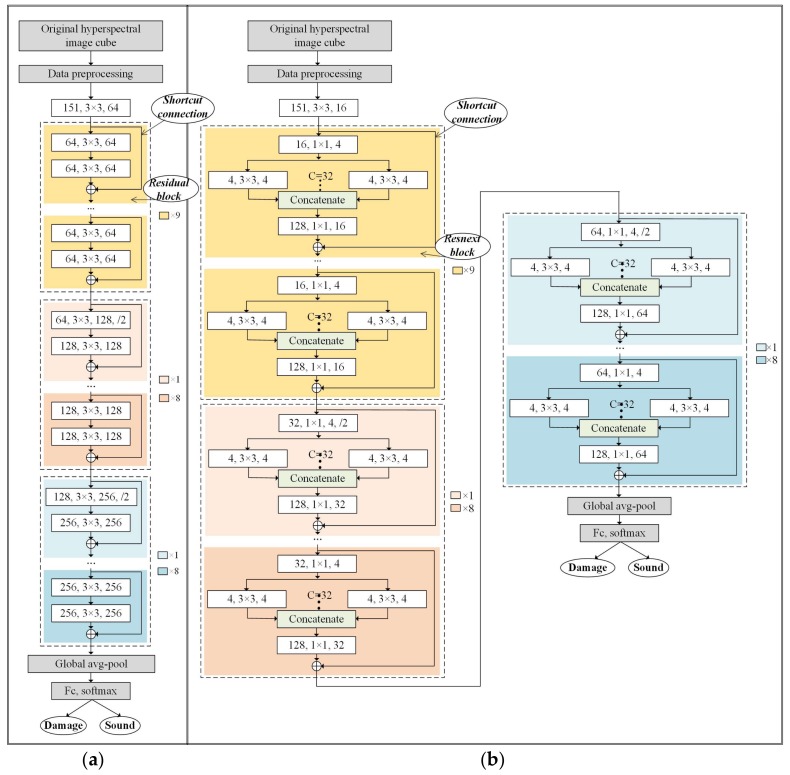
Fine-tuned architectures of ResNet (**a**) and ResNeXt (**b**) used in this study. A convolutional layer is denoted as [# input channels, filter size, # output channels, /down sampling stride]. “*C*” in (**b**) represents dividing convolutions into *C* groups.

Figure 6 shows the adjusted architectures and the fine-tuned parameters of ResNet and ResNeXt used in this study. A convolutional layer is indicated as the white rectangle, with the inside text of [# input channels, filter size, # output channels, /down sampling stride]. For example, (64, 3 × 3, 128, ./2) means: (1) the convolutional layer receives a input image with 64 channels (or 64 feature maps); (2) the filter size of this convolutional layer is 3 × 3; (3) the output of this convolutional layer contains 128 channels; (4) the output feature maps are down sampled with the stride of 2.

In ResNet (Figure 6a), preprocessed hypercubes with resolution of 32 × 32 and 151 channels are fed into the deep neural network. The first convolutional layer aims to mix the original image channels before the data enter the residual blocks. Subsequently, there are 27 Residual Blocks with different numbers of input and output channels followed by a global average pooling layer and a fully connected (FC) layers activated by softmax. The Rectified Linear Unit (ReLU) function is used as the activation function. The cross-entropy loss function along with the momentum optimizer are utilized to minimize the error. To address the overfitting issues, the batch normalization method is performed before each activation function. Learning rate is lowered as the training process progresses. The decayed learning rate is defined as:

(1) $$\mathrm{Decayed}\;\mathrm{learning}\;\mathrm{rate}\;={\;\mathrm{Learning}\;\mathrm{rate}\;*\mathrm{Decay}\;\mathrm{rate}}^{\frac{\mathrm{Global}\;\mathrm{steps}}{\mathrm{Decay}\;\mathrm{steps}}}$$

In this study, the learning rate, decay rate and decay step are set to 0.1, 0.1 and 32,000, respectively.

With respect to ResNeXt (Figure 6b), all the architecture is identical to ResNet, except the structure of ResNeXt Blocks.

The Residual Block in Figure 6a contains two convolutional layers and a shortcut connection. With the shortcut connection, the output of each layer is not the map of the inputs, but the sum of the inputs and its mappings. The shortcut connection adds the priori information to the latter layers. In the training process, reasonable prior information will promote the model performance. The ResNeXt Block in Figure 6b contains two convolutional layers, a group convolution module [29] and a shortcut connection. The group convolution module expands the number of input channels from 1 to C for each, then concatenates the results together. The number of output channels is C× # input channels.

Using k-fold cross validation, the evaluation indicators are calculated by averaging the results of 10 training processes.

### 2.4. Traditional Machine Learning Models for Blueberry Dataset

As the comparison experiments, a total of 5 traditional machine learning models, viz. Sequential Minimal Optimization (SMO), Linear Regression (LR), Random Forest (RF), Bagging and Multilayer Perceptron (MLP) are also implemented for the blueberry classification.

#### 2.4.1. Feature Extraction for Traditional Machine Learning Models

First, mask image is obtained to reduce the impact of background noise. Image at wavelength of 830.69 nm is used to get the outline mask of the sample, while the image at wavelength of 552.01 nm is used to get the reflective part mask. We receive the final mask based on morphologic processing methods.

The average transmittance spectrum is extracted as the image feature before feeding the data in the classifiers. For each spectral channel, we average the pixels at the position where the mask value is 1. Subsequently, the mean transmittance is normalized to quantize each feature to a uniform range.

**Figure 7:**
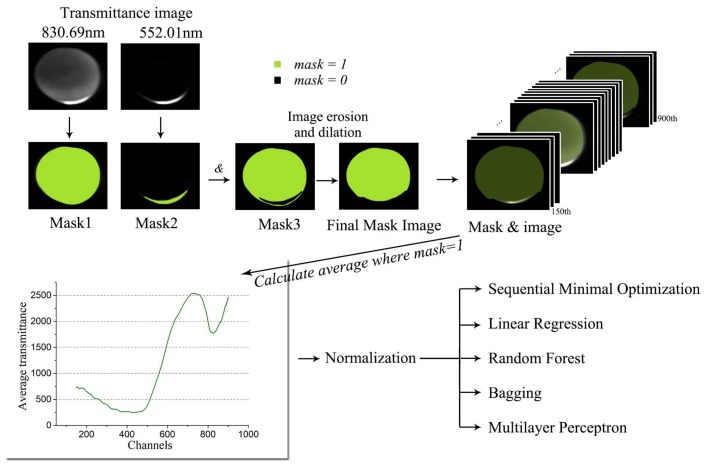
Data preprocessing and feature extraction for traditional machine learning models.

Figure 7 shows the flow of classification using traditional machine learning algorithms.

#### 2.4.2. Classifier Establishment

In this study, we choose 5 traditional machine learning algorithms as classifier. Random forest [30] uses multiple voting mechanisms for decision trees to improve decision trees in view of the shortcomings of decision trees that are easy to overfitting. Linear Regression [31] is utilized to model the relationship between a scalar dependent variable and explanatory variables (or independent variables). Sequential Minimal Optimization (SMO) [32] is a Lagrange dual problem for solving Support Vector Machine (SVM) problems. The computational cost of the traditional quadratic programming algorithm is proportional to the size of the training set, and SMO optimizes the process of solving this particular quadratic programming problem based on the characteristics of KKT conditional constraints. Bagging [33] is an approach for improving the accuracy of a learning algorithm by constructing a series of prediction functions and then combining them into a prediction function in a certain way. Multilayer Perceptron [34] is also known as full-connected neural network, using a back propagation (BP) algorithm to minimize the loss function.

### 2.5. Data Analysis

In practical applications, it is not enough to consider the accuracy of the classifier only. Because the distributors are more concerned with the situations where the classifier sorts the damaged berries as sound ones if the wrong decision is made by classifier, which will disgrace brand image, leading to greater potential economic losses than discarding the good berries. Thus, we also use recall, precision, and F1-score as evaluation indicators. These three performance metrics are seldom used by the investigators in the agricultural engineering domain. Precision is the fraction of samples classified to class c that belong to class *c* indeed, while recall is the fraction of samples in class *c* that are correctly retrieved. F1-score is an indicator used in statistics to measure the accuracy of a dichotomous model. It takes both precision and recall of classification into account, and hence can be considered as a weighted average of model precision and recall, with maximum and minimum value of 1 and 0, respectively.

(2) $$\mathrm{accuracy}=\frac{\mathrm{correctly}\;\mathrm{classified}\;\mathrm{samples}}{\mathrm{total}\;\mathrm{number}\;\mathrm{of}\;\mathrm{samples}}$$

(3) $${\mathrm{precision}}_{c}=\frac{\mathrm{samples}\;\mathrm{correctly}\;\mathrm{classified}\;\mathrm{as}\;\mathrm{class}\;c}{\mathrm{samples}\;\mathrm{classified}\;\mathrm{as}\;\mathrm{class}\;c}$$

(4) $${\;\mathrm{recall}}_{c}=\frac{\mathrm{samples}\;\mathrm{correctly}\;\mathrm{classifyied}\;\mathrm{as}\;\mathrm{class}\;c}{\mathrm{samples}\;\mathrm{of}\;\mathrm{class}\;c}$$

(5) $$F1-\mathrm{score}=\frac{1}{2}\times 2\overset{2}{\underset{c=1}{\sum }}\frac{{\mathrm{recall}}_{c}\times {\mathrm{precision}}_{c}}{{\mathrm{recall}}_{c}+{\mathrm{precision}}_{c}}$$

To visualize the performance of the classifier, we also introduce Receiver Operating Characteristics (ROC) curve, AUC (Area Under ROC Curve) and Precision-Recall curve. 

All image processing and statistical analysis were executed in Matlab R2014a (The Math Work, Inc., Natick, MA, USA). The deep learning experiment in this study was implemented using TensorFlow framework (Google Inc., Mountain View, CA, USA). A software of Waikato Environment for Knowledge Analysis (Weka) Version 3.8.2 (University of Waikato, Hamilton, New Zealand) was used to execute traditional machine learning algorithms. All experiments were performed under a Windows 10 OS on a machine with CPU Intel Core i7-7820HK @ 2.90 GHz, GPU NVIDIA GeForce 1080 with Max-Q Design, and 8GB of RAM.

## 3. Results

**Figure 8:**
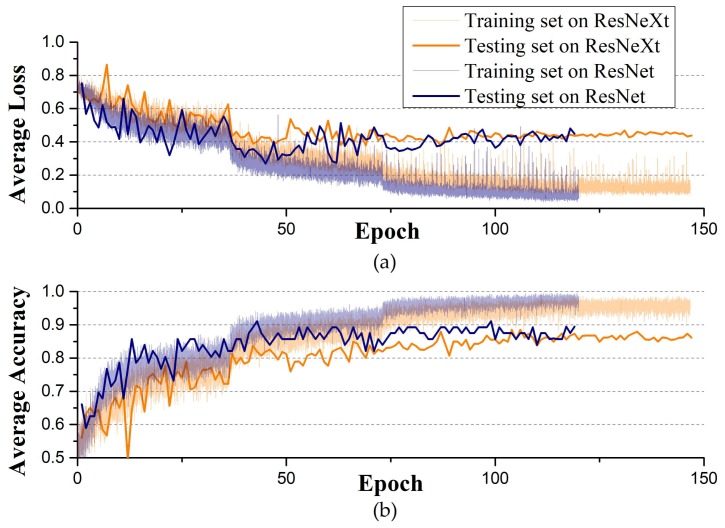
Training curves of two CNN models for blueberry damage detection. (**a**) Average loss curve; (**b**) Average accuracy.

Figure 8 shows the average loss and accuracy curves of two CNN models for blueberry damage detection. The value of loss function varies with training epochs, which constitutes the loss curve. In Figure 8a, loss curves of training set approach 0 after about 100 epochs, while the curves of testing set maintain at about 0.4 after 100 epochs. The loss curve continues to decline and stabilize, demonstrating that the model works and there is no or slightly overfitting. In Figure 8b, accuracy of training set reaches 98% after about 100 epochs, while accuracy of testing set ranges from 85% to 90%.

Table 1 gives the evaluation metrics of the two deep learning classifiers and the five traditional machine learning classifiers. K-fold cross validation (k = 10) is used in this study, and therefore all the results presented below are the average of the 10 training/testing processes.

According to the previous analysis, the cost of classifying the damaged into the sound is greater than the opposite situation. Thus, damaged berries are tagged as positive samples while the sound ones are tagged as negative samples. To sort out the damaged samples as much as possible, higher recall value is expected. In Table 1, ResNet and SMO both achieve high recall value, which is desired. However, precision of SMO is relatively low compared to ResNet. This implies that the distributor will discard excess sound berries on the results of SMO, which is unsatisfactory.

In general, the two deep learning models perform better than the traditional methods. ResNet and ResNeXt achieve extra ~8% accuracy beyond other classifiers. F1-score is the evaluation index taking both precision and recall into consideration. AUC can be used to determine the merits of the two classifiers, calculated by the area below the ROC curve. Both of the two deep learning models receive higher F1-score and AUC than the other algorithms.

**Figure 9:**
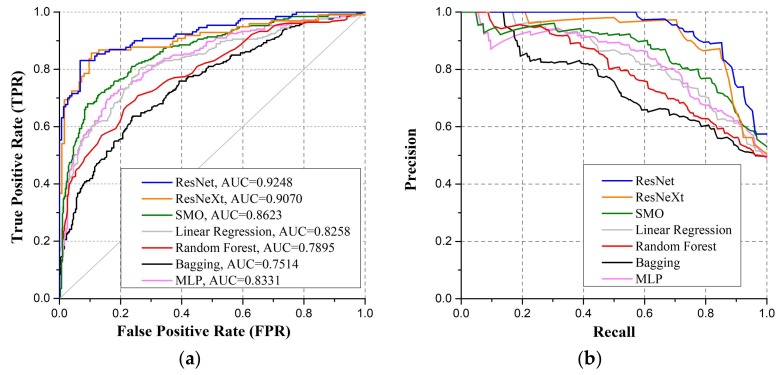
(**a**) Average ROC curves and the corresponding AUC values; (**b**) Precision-Recall curves.

In addition, we consider the ROC analysis for all the classifiers. Figure 9a shows the ROC curves and the corresponding AUC values of the all the models. A classifier generates a prediction probability for each testing sample, and then compares the predicted value with a threshold: if the probability is greater than the threshold, the corresponding sample is classified as positive class, and vice versa. The prediction probability determines the generalization of the model. Based on this probability, the testing samples can be sorted: the “most likely” positive examples are come first and the “most unlikely” come later. In this way, what the classifier does is to find a cut point in this order to divide the sample into two parts. When precision is more important, a cut point at the front position can be chosen; when recall is more significant, a cut point at back position is preferred. Thus, the reasonableness of sample sorting reflects the performance of the classifier under different tasks. The ROC curve is a visualization method that illustrates the performance of binary classifier. Samples are ordered according to the result of the classifier prediction, and different cut points are chosen in sequence. For each cut point, true positive rate (TPR) and false positive rate (FPR) are calculated respectively as the x- and y-axes. The ideal point in ROC space is the top-left corner. Curves in ROC space represent different tradeoffs as the decision boundary. AUC is an important statistical parameter for evaluating classifier performance: the closer AUC is to 1, the better overall performance of established classifier. In the current work, as shown in Figure 9a, the AUC values of ResNet and ResNeXt are 0.9248 and 0.9070, respectively, indicating that these two models achieve better performance than the other classifiers.

Figure 9b shows the Precision-Recall curves (PR-curves). PR-curve is a very widely used evaluation method in machine learning. In general, the closer the curve is to the top-right corner, the more beneficial the tradeoff it gives between precision and recall. The PR-curve in Figure 9b shows that the deep learning models can minimize the number of false positives while ensuring high classification accuracy.

The classification for each testing sample only takes 5.2 ms for ResNet and 6.5 ms for ResNeXt, which is far superior to manual sorting methods. Therefore, using configurations proposed in this study, there exists huge potential to utilize the deep learning framework for online blueberry or other fruit damage sorting.

## 4. Discussion

According to the existing literature of deep learning applied to hyperspectral images, researchers are concerned more about the application of the actual scene. However, few people are involved in the promotion of DNN architecture. Thus, we attempt to find the potential directions to improve the architecture of the deep neural network here.

Based on the above analysis, we present several ways to promote the performance of deep neural network on multi-channel images in the further study: (1) exploring algorithm to handle the correlation between channels before feeding the multi-channel images into the deep CNN; (2) implementing feature pre-extraction to standardize the samples from different production batches or places. The further explanation is presented as follows.

### 4.1. Local Correlation between Image Channels

In contrast to the natural image, the multi-channel image contains higher dimension, requiring (1) a particular method of reducing the data size to reduce the computation cost; and (2) a specific architecture of deep learning model to handle the correlation among channels. For the former problem, Ferrari et al. proposed a method for compression of hyperspectral images based on principal component analysis (PCA) without losing spectral and spatial information [35]. However, little literature is found about the latter one.

Unlike the natural color image, the hyperspectral image belongs to the multi-channel image which contains thousands of spectral channels. In the current work, although we have adjusted the CNN parameters to let the deep learning framework be suitable for hyperspectral data, the local correlation between image channels remains an unsolved problem. This problem also exists for other multi-channel image types such as computer tomography imagery.

The correlation between channels is the unignorable information when processing multi-channel images. Convolutional neural network can integrate the information between channels, which is a superior characteristic comparing to the traditional algorithms.

**Figure 10:**
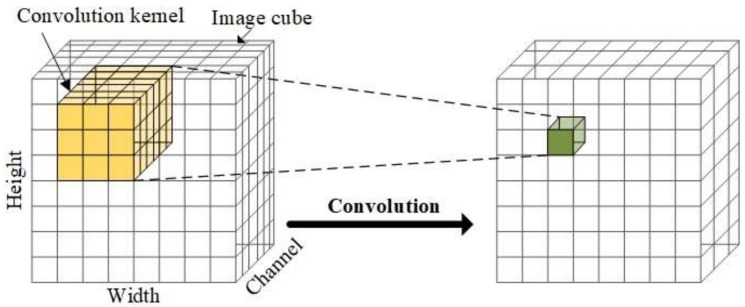
Traditional convolutional layer uses every channel of the input image, which is relatively improper for the multi-channel image.

However, as shown in Figure 10, the traditional convolutional layer uses every channel of the input data to complete the convolution operation. Such operation for multi-channel imagery does not make full use of the correlativity among channels. Also, it introduces many unnecessary parameters to be trained, which may lead to overfitting.

Image convolution is based on the local correlation in spatial dimensions, allowing the extraction of plenty of image features without training excessive undetermined weights. This idea is called “weight sharing”. Similarly, we believe that there also exists local correlation in the spectral dimension. Therefore, a specific convolutional layer for multi-channel images should be explored to handle the local correlation in all image channels for multi-channel image. In hyperspectral image case, we should design a convolutional layer that can handle the local correlation in both spectral and spatial channels.

### 4.2. Preprocessing for Hyperspectral Image

In this study, blueberries in the database only come from two production places, so the biological variation varies slightly. Also, in practical application, the production batches of submitted samples are different to those in training set. Therefore, we consider a method of feature pre-extraction to standardize the samples before training, so that the diversity of samples with various production batches or places can be reduced to some extent.

## 5. Conclusions

In this paper, we aim to implement deep CNN to classify hyperspectral blueberry samples into sound and damaged groups. Two deep convolutional neural networks (CNN), viz. ResNet and ResNeXt, are used for the detection of internal mechanical damage of blueberries based on hyperspectral transmittance data. As base-line models, a total of five traditional machine learning algorithms are also performed to complete the classification task.

Given the data structure of the hyperspectral transmittance image, hypercubes are subsampled in the spatial and spectral domain. To make the data appropriate for deep CNN training, the subsampled image hypercubes are augmented, zeros centered and normalized. We adjust the number of feature maps in the convolutional layers and the depth of the networks so that the models become applicable to the data structure of hypercube. Subsequently, we train the dataset using fine-tuned ResNet and ResNeXt models using k-fold cross validation. Besides accuracy, we also take precision, recall and F1-score as evaluation indicators to quantify false positive rate. We further plot the ROC curves and Precision-Recall curves to visualize the classifier performance. 

Fine-tuned ResNet/ResNeXt achieve average accuracy and F1-score of 0.8844/0.8784 and 0.8952/0.8905 respectively. Classifier SMO/LR/RF/Bagging/MLP obtain average accuracy and F1-score of 0.8082/0.7606/0.7314/0.7113/0.7827 and 0.8268/0.7796/0.7529/0.7339/0.7971, respectively. In practical agricultural engineering, recall is regarded as a more significant indicator, since the cost of classifying the damaged into the sound is greater than the opposite situation, and the damaged samples are expected to be sorted out as much as possible. ResNet and ResNeXt obtain recall of 0.9325 and 0.8944, respectively.

The classification for each testing sample only takes 5.2 ms and 6.5 ms respectively for ResNet and ResNeXt, indicating that the deep learning framework has a great potential for online fruit sorting.

According to the results and the problems we encountered during the experiment, we give two potential directions to fix the deficiency when analyzing multi-channel images using deep CNN in the discussion section. Overall, the classification results derived by deep learning framework are considered to be satisfying, showing the feasible prospect for the detection of blueberry internal mechanical damage in the combined use of deep CNN architecture and hyperspectral transmittance data.

## Figures and Tables

**Table 1:** Average evaluation metrics of the two deep learning models and the five traditional machine learning classifiers. The relatively best two metrics in each column are shown in bold.

Classifier	Accuracy	Recall	Precision	F1-score	AUC
ResNet	**0.8844**	**0.9325**	**0.8623**	**0.8952**	**0.9248**
ResNeXt	**0.8784**	0.8944	**0.8867**	**0.8905**	**0.9070**
SMO	0.8082	**0.9216**	0.7607	0.8286	0.8623
Linear Regression	0.7606	0.8328	0.7403	0.7796	0.8258
Random forest	0.7314	0.8031	0.7148	0.7529	0.7895
Bagging	0.7113	0.7751	0.6999	0.7339	0.7514
Multi-layer Perceptron	0.7827	0.8552	0.7523	0.7971	0.8331
